# Modeling Spatio-temporal Malaria Risk Using Remote Sensing and Environmental Factors

**Published:** 2018-09

**Authors:** Muhammad Haris MAZHER, Javed IQBAL, Muhammad Ahsan MAHBOOB, Iqra ATIF

**Affiliations:** Institute of Geographic Information Systems, School of Civil and Environmental Engineering, National University of Sciences and Technology, Islamabad, Pakistan

**Keywords:** Malaria, Climatic/environmental variables, Remote sensing, Malaria risk modeling, Pakistan

## Abstract

**Background:**

Remote sensing have been intensively used across many disciplines, however, such information was limited in spatial epidemiology.

**Methods:**

Two years (2009 & 2010) Landsat TM satellite data was used to develop vegetation, water bodies, air temperature and humidity criterion maps to model malaria risk and its spatiotemporal seasonal variation. The criterion maps were used in weighted overlay analysis to generate final categorized malaria risk map.

**Results:**

Overall, 25%, 68%, 18% and 16% of the total area of Rawalpindi region was categorized as danger zone for Jun 2009, Oct 2009, Jan 2010 and Jun 2010, respectively. The malaria risk reached at its peak during the monsoon season whereas air temperature and relative humidity were the main contributing factors in seasonal variation.

**Conclusion:**

Malaria risk maps could be used for prioritizing areas for malaria control measures.

## Introduction

Malaria is an important health problem with its endemic effects in 91 countries which consist of 40% of the world population (1). Malaria affects about 300–500 million people with 1.5 to 2.7 million deaths per year (2). Pakistan having vast irrigational system and with lot of stagnant water during monsoon season providing an ideal condition for mosquitoes breeding (3). Malaria is the second most reported disease in Pakistan (4). In 2008, 4.5 million suspected malaria cases were reported with 104454 confirmed cases of malaria. In 2009, the total reported cases in the region were 5.7 million out of which 17% cases belong to Pakistan (5). WHO in 2010 reported 6 countries with high malaria transmission in the Eastern Mediterranean Region and Pakistan was one of them.

Mosquito habitat was predicted over the coastal plain of Chiapas, Mexico. Landsat TM satellite imagery was used to classify vegetation and water bodies. They identified low, medium and high larval habitat (6). Malaria risk areas were mapped by using temperature, precipitation, and land cover maps of Thailand. Nearly 53%, 28%, and 19% areas were categorized as high, medium, and low-risk malaria region, respectively (7).

Environmental factors play an important role in epidemiology of the vector-borne diseases. Mosquitoes require suitable climatic and environmental conditions in order to complete their life cycle. Water bodies, temperature (8) forest cover (9, 10) and humidity (11, 12) are the main factors that influence malaria risk. Temperature has an immense effect on the survival and life cycle of malaria mosquitoes. At extreme temperatures (<20 °C and >40 °C), the chances of mosquitoes survival are very low. The number of days required for extrinsic cycle increases at lower temperature thus shorten the extrinsic incubation time. Higher temperature increases the extrinsic incubation period in mosquitoes. While the number of blood meals and number of times mosquitoes lay eggs increase at higher temperature (13).

Relative humidity also affects the life duration and feeding behavior of malaria mosquitoes. The life cycle of mosquitoes is shortened at low humidity (<50%) and hence there is no malaria transmission (14). The optimum humidity level for mosquito’s ranges was from 50% to 80% (15). Water bodies are necessary as these are the main breeding places for mosquitoes. Man-made dams and water pounds after heavy rainfall also provide suitable places for malaria mosquitoes breeding.

The mosquito’s flight range is an important factor in modeling malaria risk. The flight range of mosquitoes is limited to 1.2 to 1.5 km from their respective habitats. Therefore, 1.5 km area around water bodies and vegetation is considered as suitable breeding areas for mosquitoes (16, 17).

The use of remote sensing provides estimation of environmental and climatic parameters on regional basis which also accommodate the problem of variable topography. The objective of the current study was to model spatiotemporal variations of malaria risk region and compare results with malaria patient’s data provided by health department.

## Materials and Methods

### Study area

The study area Rawalpindi district is situated in northwestern part of Pakistan in the foothills of the southern slopes of north-western edges of the Himalayas. The total area of the district is 5286 km^2^ with the 4310280 population. About 53.3% of the total population lives in rural areas and 46.7% in urban areas.

The climate in the mountains is characterized as subtropical highland climate, while low lying areas have a humid subtropical climate. Different regions of district Rawalpindi i.e. Murree and Kotli Sattian experience severe winter and mild summer due to its location on higher elevation (hilly areas) while other plain regions have hot and humid summer and mild winter. The temperature ranges from 3 °C in winter to above 39 °C in summer.

### Data collection and analysis

Suspected malaria patients data of year 2009 and 2010 of four months (Jan to Apr, May to Aug and Sep to Dec) of malaria suspected cases from 131 government health centers in Rawalpindi district were collected from Directorate General Health Service (HS), Lahore. This data has been issued to the researcher by the Director General Health services through informed consent and approval of the University. Land Scan data was used for population assessment. Land Scan population dataset was downloaded from the official U.S. ORNL website (https://www.ornl.gov/) and map was generated for the study area. The weather data (2009 & 2010) was obtained from Pakistan Meteorological Department. For mapping environmental factors including vegetation cover, water bodies, temperature, and humidity, Landsat Thematic Mapper (18) was used (https://earthexplorer.usgs.gov/). Four images of 2009 and 2010 were used in this study.

### Malaria incidence mapping

Malaria incidence maps were generated by using data of suspected malaria patients collected from 131 health centers of Rawalpindi District. The formula derived for malaria incidence per 1000 persons is as follow (19);

$$\text{Malaria}\;\text{incidence}\;\text{per}\;1000\;\text{persons}=\frac{\text{No.}\;\text{of}\;\text{suspected}\;\text{malaria}\;\text{patients}}{\text{Total}\;\text{Population}}\times 1000$$

### Vegetation area mapping

Normalized difference vegetation index (NDVI) technique (NDVI varies from +1 to −1) was applied to map the vegetation cover (20).

$$\text{NDVI}=\frac{{\text{P}}_{\text{NIR}}-{\text{P}}_{\text{R}}}{{\text{P}}_{\text{NIR}}+{\text{P}}_{\text{R}}}$$

Where:

NDVI: = Normalized Difference Vegetation Index
P_NIR_: = Reflectance in Near Infrared band
P_R_: = Reflectance in Red band

### Water body mapping

Supervised classification was performed to map the water bodies in the study area (21).

A 1.5 km buffer was applied on water pixels because of the flight range of mosquitoes (16).

### Land surface temperature

Landsat TM thermal infrared data provides 120 m spatial resolution land surface temperature map. The formula for converting digital numbers into radiant temperature is as follows (22):

$$\text{T}(K)=209.831+0.834\text{DN}-0.00133\;{\text{DN}}^{2}$$

where:

T (K): = radiant temperature
DN: = digital number of analyzed pixel

For estimating land surface temperature, the emissivity correction was applied on radiant temperature. Emissivity corrected land surface temperature (23) was calculated using the following equation:

$$\text{Ts}=\frac{\text{T}(K)}{1+(\lambda \frac{\text{T}(K)}{\alpha })\text{ln}\in }-273$$

Where:

T_s_: = Land Surface Temperature in Celsius
λ: = wavelength of emitted radiance and its value is 11.5μm
∈: = emissivity=0.95
α: = h c / K = 1.438 × 10^−2^ mK
h: = Planck’s constant = 6.26 × 10^−34^Js
c: = velocity of light = 2.998 × 10^8^m/sec
K: = Stefan Boltzmann’s constant = 1.38 × 10^−23^JK^−1^

The above equation was applied on radiant temperature using raster calculator in ArcGIS to obtained land surface temperature.

### Air temperature

Two anchor pixels “cold” and “hot” pixels were selected in the Landsat TM thermal imagery for the estimation of air temperature from land surface temperature. The land surface temperature is approximately equal to near-surface air temperature at cold pixel.

dT≅0

Where:

dT: = land surface temperature − air temperature

The difference between land surface temperature and air temperature approximately equals to zero in case of cold pixel. Hot pixel is basically the barren land pixel or pixel with highest land surface temperature. The difference between land surface temperature and air temperature obtained from weather station was calculated for this pixel. The dT range was calculated by subtracting dT at cold pixel from dT at hot pixel. LST range was calculated by subtracting lowest land surface temperature from highest land surface temperature. The dT factor was calculated by dividing dT range from LST range. The difference between land surface temperature and air temperature was linear (24, 25). This linear relationship was used to convert the whole land surface temperature raster into air temperature for the study area.

### Relative humidity

The saturated water vapor pressure was calculated from air temperature maps. The formula for saturated partial water vapor pressure is as follows (26):

$${\text{e}}_{\text{s}}=6.1121\times \text{exp}\left[\left(17.502\times \text{Ta}\right){\left(240.94+\text{Ta}\right)}^{-1}\right]$$

where:

e_s_: = saturated partial water vapor pressure
Ta: = air temperature in Celsius

The partial vapor pressure was calculated from weather station data by assuming the negligible horizontal gradient of vapor pressure at the time of acquisition of images. Partial vapor pressure and saturated water vapor pressure from air temperature maps were used to calculate relative humidity. The relative humidity is the ratio of partial vapor pressure and the saturated partial vapor pressure. The formula for relative humidity is as follows (27):

$$\text{RH}=\frac{\text{e}}{{\text{e}}_{\text{s}}}\times 100$$

where:

e: = water vapor pressure

### Weighted overlay analysis

Suitability assessment for malarial risk was assessed using weighted overlay analysis based on criterion maps. Each criterion map was assigned weight according to its relative importance by using Multi-Criteria Decision Analysis (MCDA) technique. On the basis of MCDA, the weighted sum model (WSM) was developed to model the malaria risk. In this method, overlay analysis was performed by multiplying each criterion map with its relative weight and summing them together to get a risk map. According to expert’s opinion and literature review, weights of 0.35, 0.25, 0.25 and 0.15 were assigned to temperature, water bodies, humidity and vegetation, respectively (11).

$$\begin{array}{ll} \text{Malaria}\;\text{Risk}\;\text{Map} & =\{(\mathrm{Suitable}\;\mathrm{Temperature}\;\times 0.35)\\ & +\;(\mathrm{Suitable}\;\mathrm{Humidity}\times 0.25)\\ & +\;(1.5\;\mathrm{km}\;\mathrm{Area}\;\mathrm{around}\;\mathrm{Water}\times 0.25)\\ & +\;(1.5\;\mathrm{km}\;\mathrm{Area}\;\mathrm{around}\;\mathrm{Vegetation}\;\times 0.25)\} \end{array}$$

The composite layer was further reclassified into five risk classes i.e., Very High, High, Moderate, Low and No Risk Zone. Very high and high-risk zone were declared as danger zone and were addressed in further analysis. In very high risk areas, the effect of all the four criterion maps was significant and in high risk area, the effect of at least three criterion maps was significant.

### Population at risk analysis

The population at risk analysis was performed using malaria risk maps and Land Scan population database of Rawalpindi district. The overall population of study area and population in different risk classes were obtained by using zonal statistics tool in ArcGIS 9.2.

## Results

### Criterion Maps

Suitability of malaria mosquitoes was assessed based upon four environmental factors, namely air temperature, relative humidity, water bodies, and vegetation. These four data layers were used to generate malaria risk maps as several researchers used the same methodology for generation of risk maps (11, 16, 28, 29). The data layers were modified for the preparation of criterion maps with respect to their suitability. The LST of June 2009, Oct 2009, Jan 2010 and Jun 2010 were 19 °C to 43 °C, 17 °C to 42 °C, 7 °C to 29 °C and 27 °C to 52 °C respectively.

**Fig. 1:**
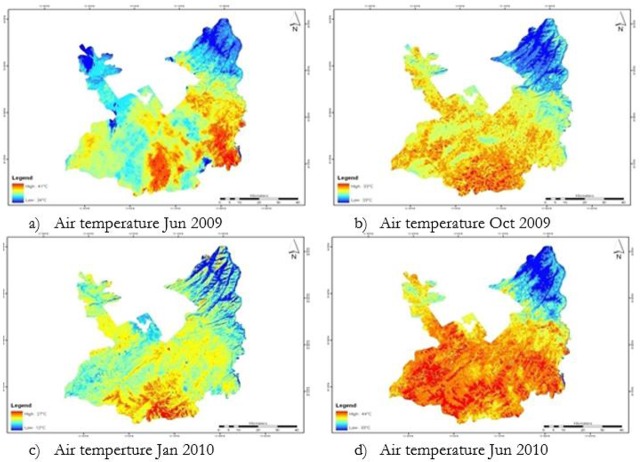
Air temperature temporal maps of Rawalpindi District

The LST maps were converted to air temperature using the meteorological observed air temperature data (Fig. 1). The derived suitable air temperature range for all life stages of malaria mosquitoes is from 25 °C to 35 °C. Reclassification was performed to extract suitable air temperature range. The suitable temperature range was assigned new value “1” and unsuitable range was assigned “0” as a new value.

**Fig. 2:**
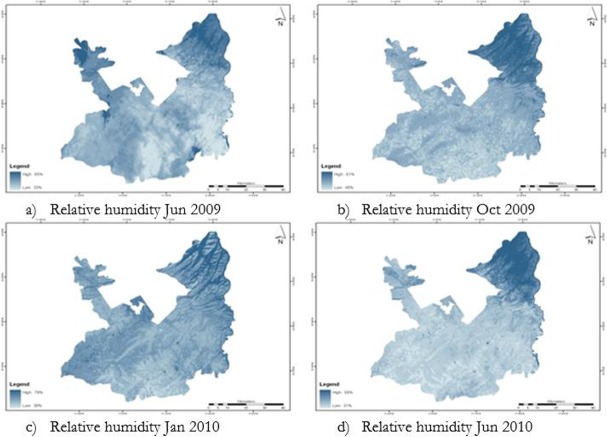
Relative humidity map of Rawalpindi District

Saturated water vapor pressure was calculated from air temperature maps. Saturated water vapor pressure along with partial vapor pressure from weather station was used to calculate air humidity maps. The relative humidity of Jun 2009, Oct 2009, Jan 2010 and Jun 2010 were 32% to 85%, 45% to 81%, 30% to 76% and 31% to 56%, respectively (Fig. 2).

Since the suitable relative humidity range is from 50% to 80% for malaria outbreak, new value of “1” was assigned to the range and rest of the pixels were assigned “0”.

The third and fourth criterion for malaria outbreak mapping was vegetation cover and water bodies present in study area. Vegetation cover and water bodies were obtained from satellite imagery using normalized difference vegetation index and supervised classification respectively.

The 1.5 km area around vegetation cover and water bodies were considered suitable because of mosquito’s flight range. Therefore, 1.5 km buffer was applied on these two criterions.

### Modeling malaria risk

**Fig. 3:**
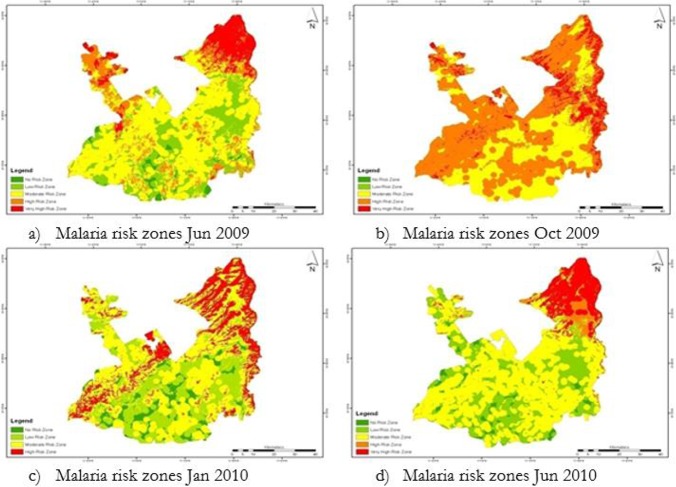
Malaria risk zonation maps of Rawalpindi District

The weighted overlay analysis was used to model malaria risk map by using environmental factors maps along with their corresponding weights. Figure 3 shows the malaria risk maps for Jun 2009, Oct 2009, Jan 2010 and Jun 2010, respectively.

### Spatio-temporal variations in trend of malaria risk area

**Fig. 4:**
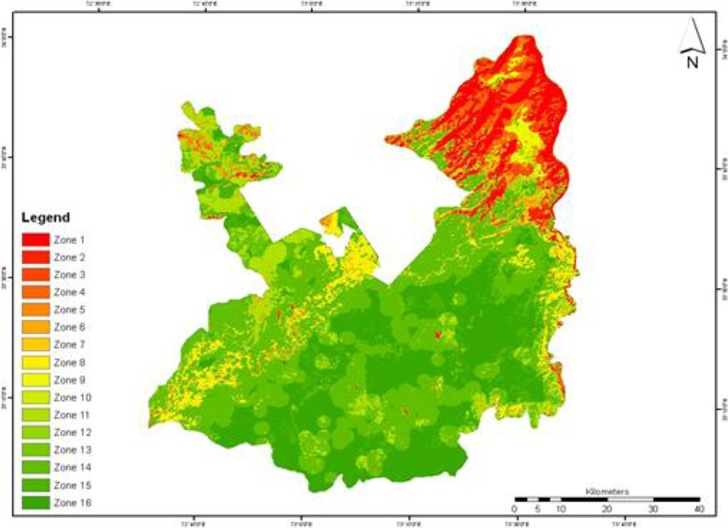
Danger zone change detection from June 2009 to June 2010

The suspected malaria patient data showed temporal and spatial trend. Fig. 4 shows the temporal changes of malaria danger zones in Rawalpindi district. The region was divided into16 zones (Table 1) whereas zone 1 shows areas having danger zone in all four images and these are the highest priority areas in Rawalpindi district.

**Table 1:** Possible month combinations along with their unique product values

***Sr. No.***	***Unique Product Value***	***Zones***	***Combinations***
1.	120	Zone 1	Jun 2009, Oct 2009, Jan 2010 and Jun 2010
2.	60	Zone 2	Oct 2009, Jan 2010 and Jun 2010
3.	40	Zone 3	Jun 2009, Jan 2010 and Jun 2010
4.	30	Zone 4	Jun 2009, Oct 2009 and Jun 2010
5.	24	Zone 5	Jun 2009, Oct 2009 and Jan 2010
6.	20	Zone 6	Jan 2010 and Jun 2010
7.	15	Zone 7	Oct 2009 and Jun 2010
8.	12	Zone 8	Oct 2009 and Jan 2010
9.	10	Zone 9	Jun 2009 and Jun 2010
10.	8	Zone 10	Jun 2009 and Jan 2010
11.	6	Zone 11	Jun 2009 and Oct 2009
12.	5	Zone 12	Jun 2010
13.	4	Zone 13	Jan 2010
14.	3	Zone 14	Oct 2009
15.	2	Zone 15	Jun 2009
16.	1	Zone 16	Danger Free Zone

Zones 2, 3, 4 and 5 show areas having danger zone in three out of four images and these areas are considered as the second highest priority areas in district. Zones 6, 7, 8, 9, 10 and 11 show areas having danger zone in two out of four images and these are the third highest priority areas. Zones 12, 13, 14 and 15 show areas having danger zone in one out of four images and these are the least priority areas. The danger zone was mostly concentrated in Murree, Kotli Sattian, Kallar Syedan and Kahuta Tehsil. In monsoon season, the risk reached its peak and each tehsil had considerable amount of danger zone during that season. Table 2 shows area of different risk classes calculated from satellite images. In October 2009, danger zone area and population at risk in danger zone were increased from 1294.61 km^2^ to 3577.39 km^2^ and 25% to 80% respectively and malaria incidence was also increased from 2.003 per 1000 person to 8.493 per 1000 person during Sep to Dec 2009. There was significant decline in danger zone and population at risk from 3577.39 km^2^ to 985.11 km^2^ and 80% to 26% respectively in Jan 2010.

**Table 2:** Risk zone areas calculated from risk maps of Jun 2009, Oct 2009, Jan 2010 and Jun 2010

***Risk Level***	***15-Jun-09***	***5-Oct-09***	***25-Jan-10***	***18-Jun-10***
	**Area (in km^2^)**			
Very High Risk Zone	517.11	453.19	0	591.84
High Risk Zone	777.50	3124.20	985.11	267.15
Moderate Risk Zone	2768.21	1717.81	2584.93	2962.18
Low Risk Zone	1121.66	6.63	1356.13	1340.19
No Risk Zone	116.64	2.23	372.95	150.44

**Fig. 5:**
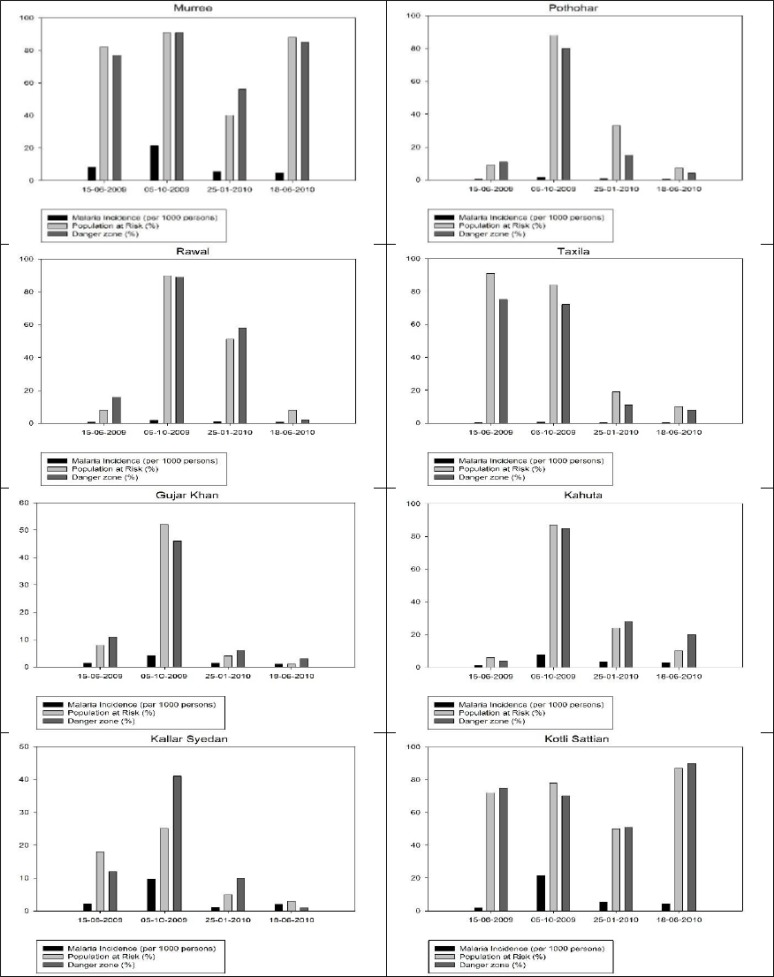
Tehsil wise percentage of danger zone and population at risk in Rawalpindi

This result was in agreement with malaria incidence data which abruptly dropped from 8.493 per 1000 person to 2.307 per 1000 person during that period. In Jun 2006, there was slight decrease in danger zone from 985.11 km^2^ to 858.99 km^2^ and malaria incidence was also decreased from 2.307 per 1000 person to 2.018 per 1000 person. Fig. 5 shows the percentage of danger zone and percentage of population lives in danger zone along with malaria incidence at that time in Muree, Kotli Sattian, Kahuta, Kallar Syedan, Gujar Khan, Pothohar, Rawal and Taxila tehsil respectively. The first four tehsils including Murree, Kotli Sattian, Kahuta and Kallar Syedan were facing high degree of risk throughout the year. The maximum danger zone was observed in Oct 2009.

This high degree of risk was not observed in other four tehsils of district due to lack of suitable climatic condition in these regions. These areas experienced high danger zone only in Oct 2009.

Malaria distribution maps generated by populating malaria suspected patient data in Tehsil boundaries of Rawalpindi district revealed that the overall malaria incidence in Rawalpindi district was highest in monsoon season and least in winter season.

The temporal analysis of results indicates that danger zone area was increased from 1294.61 km^2^ to 3577.39 km^2^ from Jun 2009 to Oct 2009. This increase could be due to suitable humidity and temperature for mosquitoes in October 2009.

## Discussion

The strength of this study is that the combination of ground data, remote sensing data and GIS spatial modeling techniques aided in the identification and spatial distribution of malaria severity and malaria risk zones in Rawalpindi region. The current study is one of the first to identify malaria environmental risk factors in a region with high malaria reported cases. The remote sensing approach can readily be used on regional scales in other parts of the country to guide targeted malaria control interventions to further reduce malaria transmission.

Air temperature and relative humidity were the main contributing factors in seasonal variation of malaria risk areas. While the contribution of water bodies and vegetation cover in seasonal variation of malaria risk area was low. Water bodies and vegetation are perineal in nature and mostly present in the mountainous region of the study area. There is no doubt that water body plays a significant role in the life cycle, development and prevalence of malaria mosquitoes but in this study their contribution in the seasonal variation of malaria risk area is not as significant as compared to the presence of suitable temperature and relative humidity.

Some studies suggest the presence of water bodies as the major contributing factors in the designation of malaria risk area (30). These studies were conducted in tropical areas with large number of water bodies/ponds compared to our study area. Another reason could be due to large spatial variation of air temperature and relative humidity across a region with significant variation in topography (southern plain & Himalayan Mountains in the northern part of the district Rawalpindi). Remote sensing has been used in other studies for identification of malaria risk areas in other regions of the world. Some identified factors include water bodies, and dam (31). In French Guiana meteorological and landscape data were used in mapping the density of Anopheles mosquitoes (32). In Dar es, Salaam higher malaria risks were associated with nearness to dense vegetation, inland water, and wet/swampy areas while lower risk of infection was predicted in densely built-up areas.

Relationship between environmental variables estimated by remote sensing and the spatial distribution of seven mosquito species vectors of West Nile and other pathogens was established (33). However, none of these studies used such an exhaustive set of remote sensing estimated environmental factors and ground data for mapping the malaria risk and its monthly temporal variations. A strong association was found between malaria patient data (malaria incidence per 1000 persons) obtained from the health department, danger zone (percent) map and population at risk (percent) map (Table 3).

**Table 3:** Correlation between reported (n=32) malaria incidents (per 1000 person) population at risk (%), and danger zone (%)

***Variables***	***Malaria Incidence (per 1000 persons)***	***Population at Risk (%)***
Malaria Incidence (per 1000 persons)	1	
Population at Risk (percentage)	0.43^**^	1
Danger Zone (percentage)	0.47^**^	0.98^**^

** Significant at 0.01 probability level

For more accurate mapping of malaria risk areas, high-resolution satellite imagery should be used for mapping vegetation and water bodies.

## Conclusion

Temperature and relative humidity were found to be the main contributing factors that influence malaria risk. In addition, temperature and humidity also significantly contribute to seasonal variation of malaria risk areas. Remote sensing and GIS technology provided an efficient and inexpensive method of mapping malaria risk area using Landsat TM imagery on fortnight basis. The finding of this study could be used by policymakers and planner to target priority areas with control activities (insecticide house spraying, larviciding).

## Ethical considerations

Ethical issues (Including plagiarism, informed consent, misconduct, data fabrication and/or falsification, double publication and/or submission, redundancy, etc.) have been completely observed by the authors.
